# Radiographic and MRI Assessment of the Thrower’s Elbow

**DOI:** 10.1007/s12178-021-09702-x

**Published:** 2021-04-17

**Authors:** G. M. Powell, N. S. Murthy, A. C. Johnson

**Affiliations:** Department of Radiology, Mayo Clinic, 200 First St. SW, Rochester, MN 55905 USA

**Keywords:** Elbow, Thrower’s elbow, Overhead throwing athlete, MRI

## Abstract

**Purpose of Review:**

Throwing athletes are vulnerable to elbow injuries, especially in the medial elbow, related to high stress and valgus load in both acute and chronic settings as a result of this complex biomechanical action. This current review details the relevant anatomy and imaging features of common elbow pathology identified with radiographs and MRI in throwing athletes.

**Recent Findings:**

Although elbow pathology in throwing athletes is well documented, advances in imaging technology and technique, particularly with MRI, have allowed for more detailed and accurate imaging description and diagnosis.

**Summary:**

Pathology of thrower’s elbow occurs in predictable patterns and can be reliably identified radiologically. Clinical history and physical examination should guide radiologic evaluation initially with radiographs and followed by an MRI optimized to the clinical question. Constellation of clinical, physical, and radiologic assessments should be used to guide management.

## Introduction

Throwing athletes are particularly prone to elbow injuries and include baseball, softball, football, and javelin throwers. Baseball pitchers experience higher rotational and angular velocities compared to other throwing sports [1]. The throwing motion is a complex biomechanical action which is most extensive in baseball pitchers and is divided into six phases: windup, early cocking, late cocking, acceleration, deceleration, and follow through [2]. The elbow is exposed to high stress and valgus load during the later phases of throwing which can often exceed the inherent strength of medial stabilizers. Elbow injuries in throwing athletes are typically related to valgus load resulting in acute and chronic overuse injuries [3, 4]. Preoperative radiologic evaluation with radiographs and subsequent magnetic resonance imaging (MRI) allow for accurate diagnoses which help guide management. The purpose of this manuscript is to review imaging features of common elbow pathology identified with radiographs and MRI in throwing athletes.

## Technique

Radiographic evaluation of the elbow should routinely be performed prior to advanced imaging to assess for abnormalities including malalignment, fracture, arthrosis, osteochondral defects, osteocartilaginous bodies, joint effusion, and soft tissue calcification. Anteroposterior and lateral radiographs are performed with the elbow extended and the forearm supinated and the elbow flexed to 90° with the forearm pronated, respectively. Tube angle is perpendicular to the image receptor with source to image distance of 48 inches and exposure parameters of 55 kVp and 4 mAs. If clinically warranted, oblique, flexion, extension, gunslinger lateral, radial head, and cubital tunnel projections can also be obtained for a more sensitive evaluation. Occasionally, radiographic or fluoroscopic stress views are used to assess ligament integrity and joint instability. Stress views may be beneficial in the setting of high clinical suspicion of instability and an equivocal or nondiagnostic MRI.

MRI evaluation of the elbow should be performed at field strengths no less than 1.5T; 3.0T is preferred due to higher spatial resolution. Patients are either placed in the prone “superman” or supine position with the arm at the side (forearm supinated and elbow extended). The latter position allows for a more accurate assessment of the anterior bundle of the ulnar collateral ligament (UCL) which is taut in extension. Alternatively, elbow flexion would be preferred to interrogate the integrity of the posterior band of the UCL. Image quality and field homogeneity are optimized in the “superman” position as the elbow is closer to the isocenter of the magnet. Phased array surface and circumferential coils are used to obtain an appropriate signal to noise ratio and adequate signal from the tissue volume, respectively [5–7].

Comprehensive multiplanar evaluation of the elbow includes T1-weighted or proton density and fluid-sensitive sequences, with at least one plane of T1-weighting, acquired in the axial, coronal, and sagittal planes. Inversion recovery sequences should replace fat-saturated fluid sensitive sequences to minimize magnetic field inhomogeneity in the setting of off-center positioning of the elbow relative to the magnet’s isocenter [7].

High-resolution (512–384 × 256 matrix, 3-mm slice thickness) intermediate echo time fast spin echo (FSE) sequences are acquired in the coronal plane to reduce partial volume averaging and evaluate ligaments, tendons, and cartilage. The remainder of the elbow structures is thoroughly evaluated with high-resolution (512–384 × 256 matrix, 3–4-mm slice thickness) intermediate echo time FSE sequences acquired in the axial and sagittal planes. In skeletally immature patients, fat-suppressed gradient-recalled echo (GRE) is appropriate to characterize cartilage of unfused physes.

Although some authors prefer MR arthrography with intraarticular gadolinium-based contrast or saline to facilitate recognition of partial tears of closely apposed ligaments, in our experience, high-resolution noncontrast MRI is sufficient for diagnosing clinically relevant pathology [8–10]. Additionally, in the setting of MR arthrography, cartilage and synovial assessment can be confounded by adjacent hyperintense signal and joint capsule distention, respectively.

## Relevant Imaging Anatomy

The UCL is comprised of anterior, posterior, and transverse bundles. The UCL serves as the primary restraint to valgus stress, particularly the anterior bundle. Originating on the undersurface of the medial epicondyle, the cord-like anterior band gently tapers and inserts within 2 mm of the sublime tubercle as it merges with the ulnar periosteum [6, 11, 12]. Although biomechanically the anterior bundle is divided into anterior and posterior bands, these are not typically appreciated as distinct structures on imaging [13–15]. The fan-shaped posterior bundle appears as a capsular thickening forming the floor of the cubital tunnel extending from the undersurface of the medial epicondyle to the margin of the trochlear notch [16, 17]. The transverse bundle extends from the medial olecranon to the inferomedial coronoid process. The transverse bundle does not significantly contribute to valgus stability. As a result of routine MRI positioning of the elbow in extension, the posterior and transverse bundles are not routinely assessed.

The UCL is best evaluated in the coronal plane. Ligament morphology is assessed with GRE and FSE sequences, whereas signal intensity is assessed with inversion recovery and FSE sequences. Occasionally, the origin of the anterior bundle of the UCL can have a slightly striated appearance as a result of interdigitating fat; otherwise, the ligament is normally homogenously hypointense on all pulse sequences [11, 18]. Deep to the origin of the posterior band of the anterior bundle, there is a prominent synovial fold which should not be interpreted as a tear [3, 8, 19]. The UCL is in close apposition to the overlying deep muscle fibers of the flexor digitorum superficialis.

The flexor-pronator muscle complex originates from the medial epicondyle and is comprised of the pronator teres, flexor carpi radialis, palmaris longus, flexor digitorum superficialis, and flexor carpi ulnaris muscles with various distal attachments. Additionally, the flexor digitorum superficialis, flexor carpi ulnaris, pronator teres, and brachialis comprise the flexor-pronator mass [20]. This muscle group provides dynamic stability to valgus stress. Similar to the UCL, the flexor-pronator mass tendons are normally homogeneously hypointense on all pulse sequences. Like with ligaments, tendon morphology is assessed with GRE and FSE sequences, whereas signal intensity is assessed with inversion recovery and FSE sequences. The flexor-pronator mass is best evaluated in the axial and coronal planes.

The ulnar nerve courses through the arcade of Struthers approximately 8cm proximal to the elbow, along the medial aspect of the distal triceps, passes posterior to the medial epicondyle, and through the cubital tunnel [21, 22]. The cubital tunnel floor is formed by the posterior bundle of the UCL and joint capsule, whereas the roof is formed proximally by the cubital tunnel retinaculum (Osborne ligament) and distally by the flexor carpi ulnaris aponeurosis (arcuate ligament) [23]. Occasionally, an anomalous anconeus epitrochlearis may overlie the cubital tunnel. The ulnar nerve commonly has no brachial branches and rarely provides innervation to the triceps [22]. Innervation to the flexor carpi ulnaris is typically via the first motor branches of the ulnar nerve as it courses between the muscular heads superficial to the anterior bundle of the UCL after exiting the cubital tunnel. Given the anatomic location of the ulnar nerve, it is exposed to traction forces related to valgus stress and flexion, as well as nerve compression [21, 23].

The ulnar nerve is best evaluated in the axial plane. The isointense uniform fascicular morphology of the ulnar nerve is assessed with GRE and FSE sequences. The signal intensity of the ulnar nerve is normally isointense to slightly hyperintense when assessed with inversion recovery and FSE sequences. The size of the ulnar nerve should be maintained or gently taper as it courses about the elbow distally.

When evaluating musculoskeletal structures, it is imperative to understand technical limitations, which could be misdiagnosed as pathology. Although it is outside the scope of this article, recognition of imaging artifacts such as magic angle artifact is critical for interpretation. Magic angle artifact is most evident in sequences with a short echo time (TE) such as T1-weighted, PD, and GRE sequences and is increasingly diminished with longer TE sequences such as T2-weighted sequences. Magic angle artifact results in an artefactual increase in signal intensity within highly organized tissue when it is oriented at approximately 55° to the main magnetic field. Anatomic structures subject to this artifact include tendons, ligaments, and peripheral nerves. It is important not to misinterpret magic angle artifact as pathology when evaluating these structures.

## UCL Injuries

As the primary static stabilizer of the medial elbow, the UCL provides 54% of the total resistance against valgus loading in flexion predominately relying on the anterior bundle between 30 and 120° [24•]. The posterior bundle of the UCL contributes to valgus resistance at elbow flexion angles greater than and equal to 120° [25]. The elbow is flexed 30–90° in the typical throwing motion which can generate valgus torque and medial tensile force that exceeds the inherent stability of the ligament; however, secondary stabilizers provide the necessary biomechanical support to prevent medial elbow failure [24•, 25]. Alternative methods of throwing (e.g., sidearm or submarine) and poor mechanics can lead to even greater forces across the ligament [24•].

**Fig. 1:**
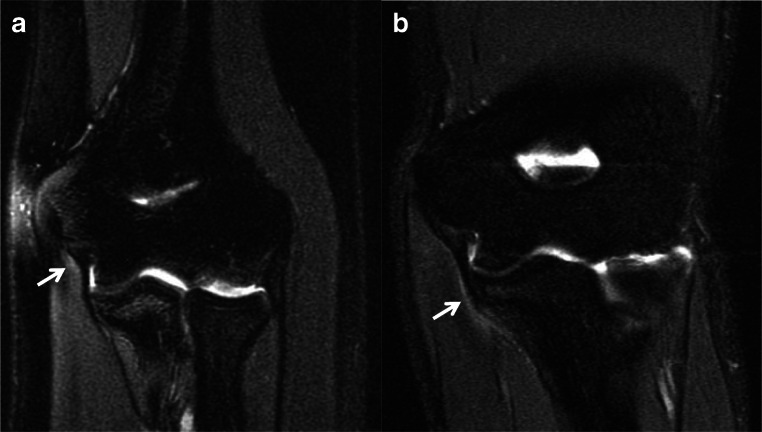
Coronal T2 MR images of the elbow in different patients demonstrate periligamentous edema about the proximal (**a**, arrow) and distal (**b**, arrow) UCL consistent with low-grade sprains

Once the inherent strength is exceeded, ligamentous injury results. Acute injuries to the UCL can manifest as altered signal intensity, laxity, discontinuity, and reactive changes in the periligamentous soft tissues. Periligamentous edema with otherwise intact ligament fibers is consistent with a low-grade sprain in the appropriate clinical setting (Fig. 1) [23]. In the absence of a well-defined partial thickness tear, intrasubstance signal abnormality, ligament attenuation, and laxity represent interstitial microtears referred to as interstitial load [26].

**Fig. 2:**
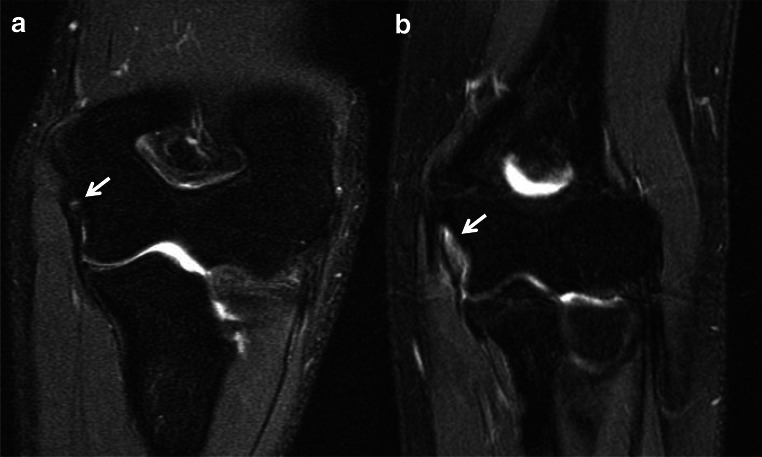
Coronal T2 MR images of the elbow in different patients demonstrate a low- (**a**, arrow) and high-grade (**b**, arrow) partial thickness tears of the UCL

Fiber discontinuity is consistent with tear. The UCL humeral origin is the most common location of a tear, whereas midsubstance and distal tears are least common [5]. Partial tears are classified as high grade or low grade based on greater or less than 50% involvement of the ligament thickness, respectively. Although partial thickness tears can be identified with fluid signal interposed between disrupted ligament fibers, more commonly they are diagnosed in the setting of ligament hyperintensity and indistinctness (Fig. 2) [26]. Additionally, a distal partial thickness tear can be identified when fluid insinuates between the deep fibers of the ligament which have stripped off from the sublime tubercle insertion [8]. The “T-sign” describes this appearance, which is seen with nonarthrographic and arthrographic MRI evaluations. Although the sensitivity of nonarthrographic MRI to evaluate partial thickness tears has been called into question, more recent studies have reported higher sensitivities when examining with high-resolution fluid sensitive intermediate echo time FSE sequences [6, 8, 27].

**Fig. 3:**
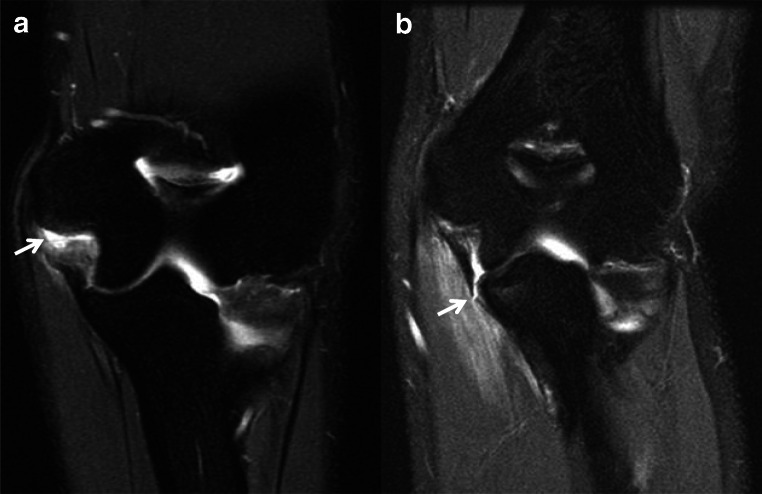
Coronal T2 MR images of the elbow in different patients demonstrate proximal (**a**, arrow) and distal (**b**, arrow) full thickness tears of the UCL

A full thickness tear is present when there is complete fiber disruption which can be identified with a combination of findings such as increased signal intensity and fiber indistinctness, laxity, or discontinuity in the absence of any distinctly intact fibers (Fig. 3). Additionally, an avulsion fracture of the sublime tubercle or traction osteophytes at the attachment of the UCL anterior bundle is functionally equivalent to complete disruption as this ligament no longer provides a functional valgus restraint to the medial elbow [27].

**Fig. 4:**
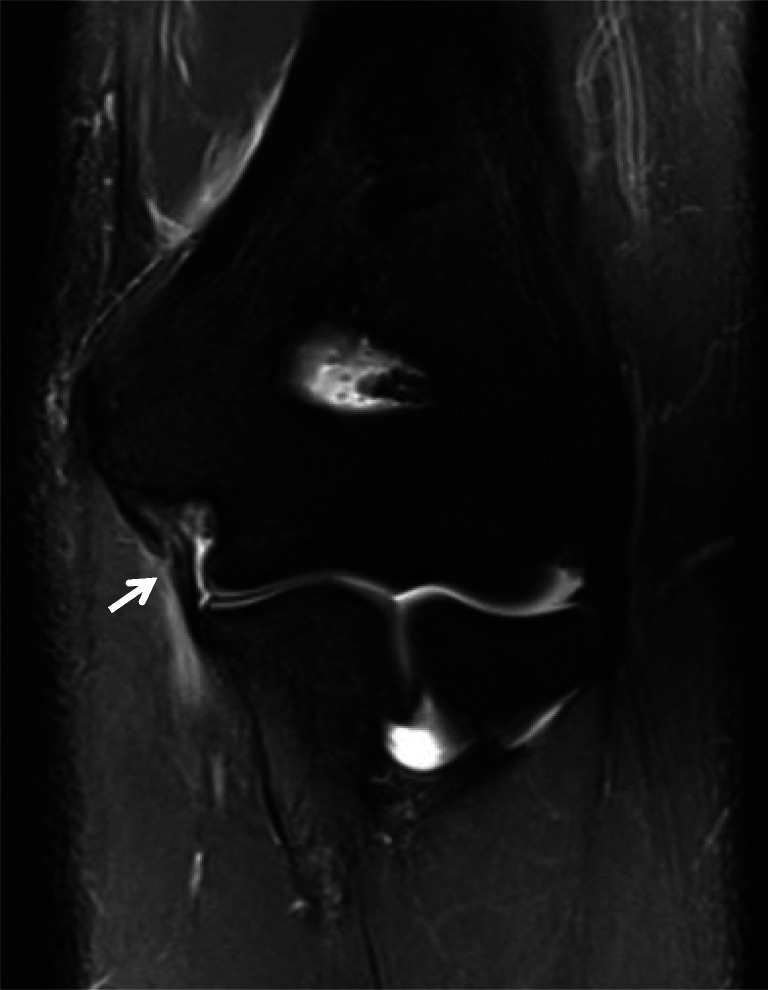
Coronal T2 MR arthrogram of the elbow demonstrates a high-grade partial thickness tear of the UCL midsubstance consistent with a 2A injury (Ramkumar classification system, arrow)

Ramkumar et al. have proposed a 6-stage MRI-based classification system for UCL injuries to aid in preoperative decision-making [28]. The classification system takes into consideration the location and degree of tear. The location of the tear is classified as proximal (1), midsubstance (2), or distal (3) UCL and degree of the tear as partial (A) or complete (B) (Fig. 4). Substantial to near perfect intraobserver and interobserver reliability has been demonstrated and this classification system can facilitate consideration of operative versus nonoperative management [28, 29].

**Fig. 5:**
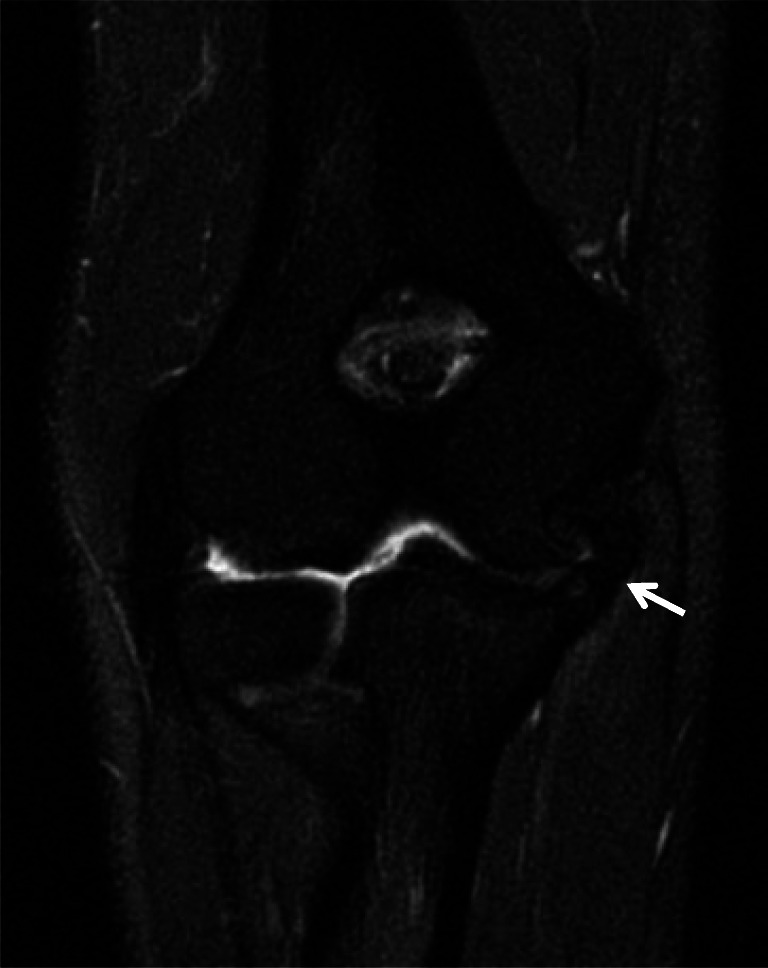
Coronal T2 MR image of the elbow demonstrates chronic low signal thickening of the UCL (arrow)

Overuse and repetitive stress can result in chronic remodeling of the UCL. Even in the asymptomatic thrower, asymmetric ligament thickening and altered signal intensity have been identified (Fig. 5) [5, 30]. A chronically stressed UCL may also have mildly increased hyperintensity on fluid sensitive sequences (representing chronic microtears), laxity, and/or redundancy [31–33]. Additionally, intraligamentous calcification and heterotopic ossification can be identified radiographically and with MRI representing sequelae of a remote or chronically injured UCL [26]. Reactive bone marrow edema-like signal at the UCL humeral or ulnar footprints can result from acute and chronic stress reaction related to traction.

## Chronic Valgus Overload

**Fig. 6:**
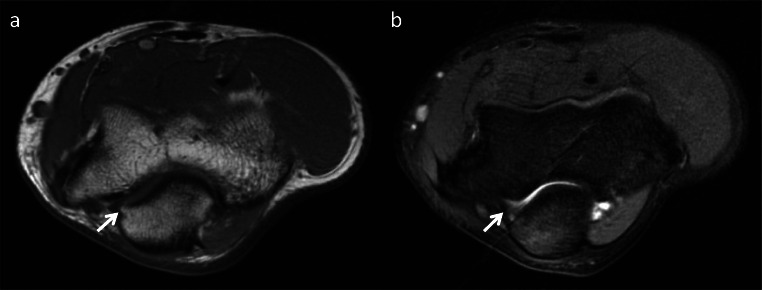
**a** Axial T1 MR image of the elbow demonstrates a small osteophyte along the posteromedial olecranon (arrow). **b** Axial T2 MR image demonstrates posteromedial ulnohumeral synovitis (arrow), mild pericapsular edema, and bone marrow edema-like signal in the olecranon

During the late acceleration and follow-through phases of throwing, there is a large mechanical force and load on the posteromedial elbow [22, 25]. Repetitive chronic valgus stress and overload can result in UCL attrition and, ultimately, failure [26]. Secondary posteromedial impingement and osteoarthrosis are most evident in the posteromedial joint space. Posteromedial synovitis as a result of posteromedial impingement is most conspicuous on sagittal and axial FSE sequence represented by synovial and capsular thickening and irregularity with or without pericapsular inflammation (Fig. 6). The associated osseous remodeling initially includes subchondral sclerosis particularly at the posteromedial ulna and abutting posterior trochlea. As posteromedial impingement progresses, chondral thinning in the posteromedial ulnohumeral articulation can lead to osteophyte formation especially on the posterior and medial olecranon [30]. Osteocartilaginous bodies can result from chondral injury and osteophyte fragmentation or fracture. Additional signs of posteromedial impingement as related to osteophyte formation or osteocartilaginous bodies can present with the inability to fully extend the elbow for standard image positioning.

## Medial Epicondylosis

**Fig. 7:**
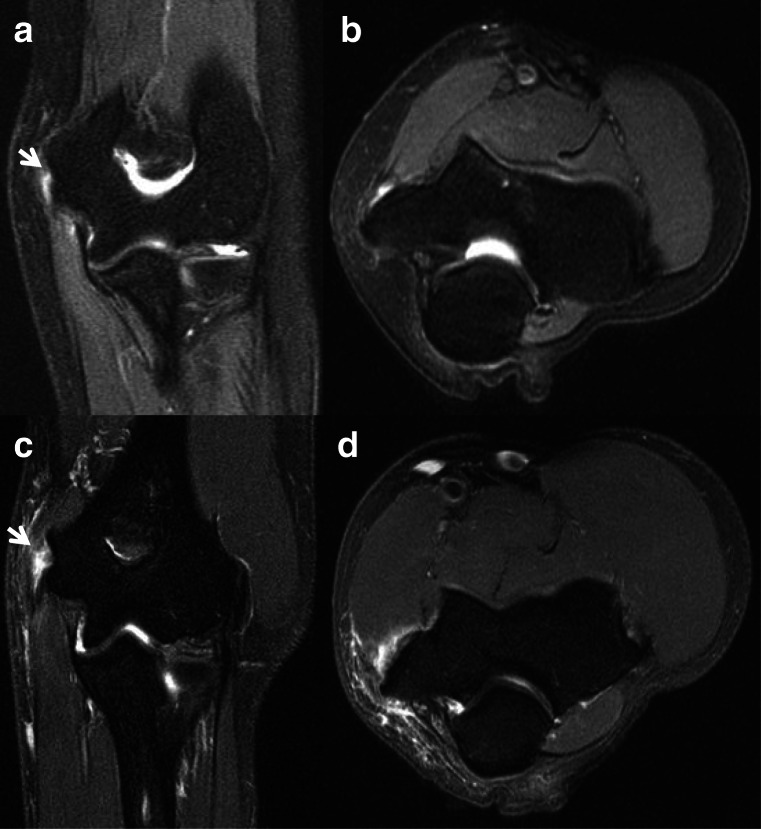
Coronal (**a**) and axial (**b**) T2 MR images of the elbow in the same patient demonstrate medial epicondylosis with low-grade partial thickness tears (arrow) and an adjacent UCL sprain. Coronal (**c**) and axial (**d**) T2 MR images of the elbow in a different patient demonstrate medial epicondylosis with high-grade partial thickness tears (arrow) and adjacent chronic thickening of the UCL

The flexor-pronator muscle complex provides dynamic stability to the medial elbow offloading the stress to the UCL [25]. This muscle group is activated in the acceleration phase of throwing and during forceful wrist flexion as the ball is released [21]. Specifically, the pronator teres and flexor carpi radialis are the most activated components during the acceleration phase and are also the most commonly injured in medial epicondylosis [3, 24•, 34]. Medial epicondylosis in the setting of chronic valgus overload manifests as tendinosis and tears. Intermediate to increased T2 signal intensity within the tendon substance corresponds to collagen disruption, tendon degeneration, and neovascularization with or without focal enlargement represents tendinosis [35]. Partial or full thickness tears demonstrate fluid signal between tendon fibers with associated fiber discontinuity and/or indistinctness (Fig. 7). In addition to these injuries, an acute valgus load to the elbow can result in contusion and extensive soft tissue edema [4]. Calcific tendinosis can also occur in the flexor-pronator complex manifesting as amorphous calcification at the medial elbow radiographically and an intratendinous hypointense nidus with adjacent edema on fluid sensitive sequences. Chronically degenerated or previously injured tendons can develop areas of heterotopic ossification or dystrophic calcifications. In the setting of medial epicondylosis, up to 60% of patients can have associated ulnar neuropathy [36].

## Ulnar Neuropathy

Ulnar neuropathy is common among throwing athletes as a result of concurrent medial elbow injury or in isolation [24•]. Types of injuries include traction, friction related to subluxation, and compression [25]. Chronic traction as a result of excessive valgus laxity is most common cause of ulnar nerve injury in throwing athletes [21, 26]. Compression can occur at a number of sites and could be related to osteophytes, muscle hypertrophy, accessory and low lying muscles, fascial and ligamentous thickening, ganglia, and masses [23, 37].

**Fig. 8:**
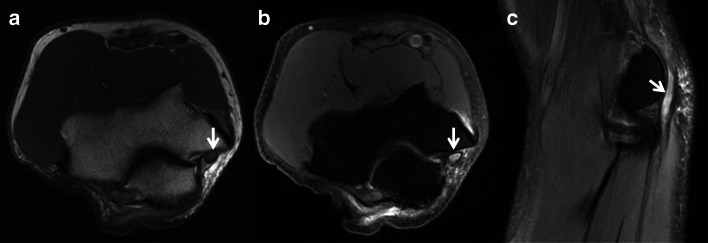
Axial T1 (**a**) and axial (**b**) and coronal (**c**) T2 MR images of the elbow demonstrate ulnar nerve (arrows) enlargement, loss of the normal fascicular architecture, and T2 signal hyperintensity consistent with ulnar neuritis

Ulnar neuropathy may manifest as nerve or fascicular enlargement with or without loss of the normal fascicular architecture proximal to or within the cubital tunnel (Fig. 8). Hyperintense signal intensity can be identified on fluid sensitive sequences; however, this can also be seen in asymptomatic patients. Therefore, these findings are sensitive, but not specific for neuropathy and must be interpreted in the appropriate clinical setting [23].

## Osteochondral Lesions

**Fig. 9:**
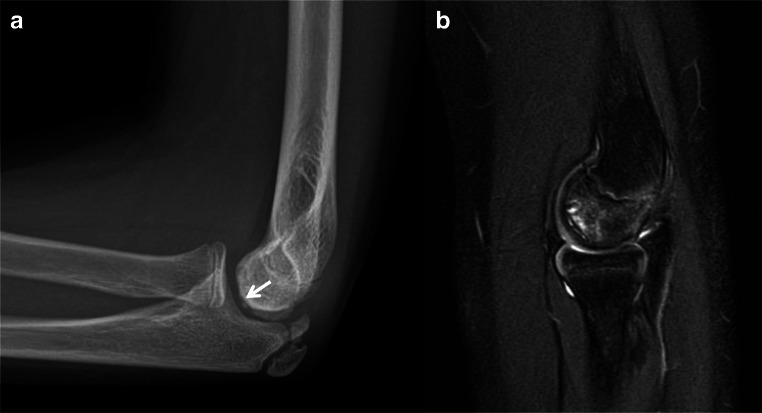
**a** Lateral radiograph of the elbow demonstrates osseous irregularity and sclerosis of the capitellum with subtle fragmentation (arrow). **b** Sagittal T2 MR image more clearly demonstrates collapse of the capitellar subchondral bone plate with subjacent cyst-like changes consistent with an osteochondral lesion

The etiology of osteochondral defects is multifactorial and primary osseous vascular insufficiency as a result of altered biomechanics and repetitive trauma with secondary involvement of the overlying cartilage is most likely [31, 38••]. Osteochondral injury can occur during an acute valgus load, direct impaction, or develop as a result of chronic valgus overload. Osteochondral lesions in throwing athletes occur most commonly in the capitellum; however, the radial head, trochlea, lateral trochlear ridge, olecranon, and olecranon fossa have also been reported [23, 39, 40]. Radiographically, osteochondral lesions can be identified as a curvilinear lucency undercutting subchondral bone and articular surface incongruity with or without osseous fragmentation and osteocartilaginous bodies (Fig. 9). Subtle subchondral flattening and mildly increased chondral hyperintensity on fluid sensitive sequences can represent evidence of an early osteochondral lesion; this was previously referred to as osteochondritis dissecans [41]. Additional osseous findings include a focal area of hazy subchondral T1 hypointensity and corresponding T2 hyperintensity or a more defined focal area of T1 hypointensity with a peripheral rim of T2 hyperintensity [39]. As these changes progress, there may be fragmentation, collapse, or cystic resorption of the subchondral bone plate. Lesions are also assessed for stability and an unstable fragment is indicated by fluid signal or cystic change interposed between the fragment and parent bone, sclerosis, fragmentation, and disruption of the overlying cartilage [38••, 39]. Various MRI staging systems exist for assessing the stability of osteochondral lesions in the elbow [42–44].

## Medial Epicondyle Apophysitis

**Fig. 10:**
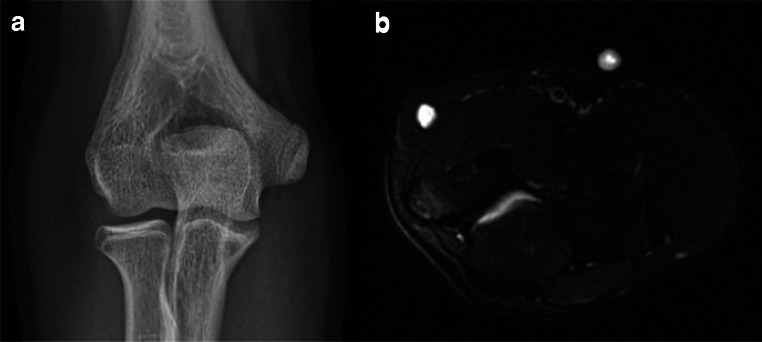
**a** Anteroposterior radiograph of the elbow in a patient with prolonged pain demonstrates a bulbous medial epicondyle apophysis. **b** Axial T2 MR image demonstrates bone marrow edema-like signal in the medial epicondyle apophysis and increased signal in the physis with adjacent soft tissue edema. Findings are consistent with medial epicondyle apophysitis

Acute and chronic stresses to the medial elbow in skeletally immature athletes are preferentially transmitted to the biomechanically weaker medial epicondylar apophysis rather than the UCL itself. This process is typically referred to as “little league elbow” [25, 45]. In the acute setting, this may manifest as a physeal (Salter-Harris I) fracture with variable distraction, irregularity, and sclerosis [21, 25]. Physeal widening can also be seen in traction apophysitis with or without fragmentation of the medial epicondylar apophysis [5]. Associated bone marrow edema-like signal is easily identified on fluid sensitive sequences and can be seen with both injury patterns; therefore, differentiation is often dependent on clinical history (Fig. 10). Sequelae of a physeal injury prior to fusion may present as a more bulbous appearing medial epicondyle [26].

## Fracture

**Fig. 11:**
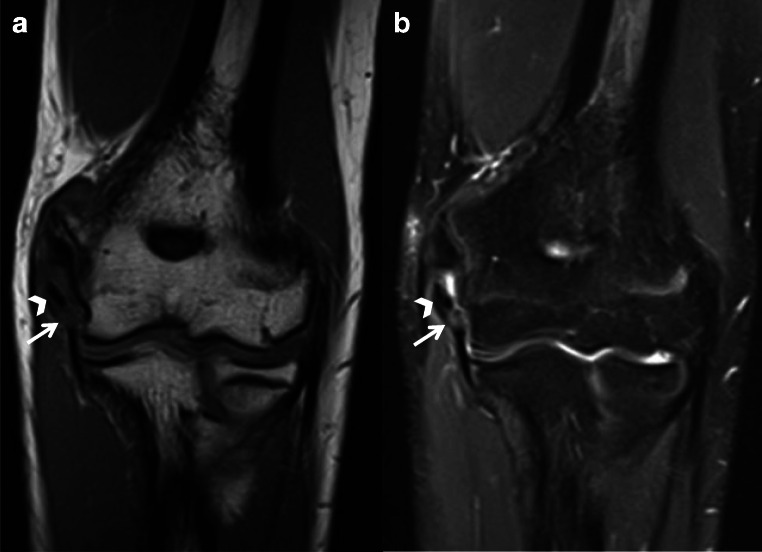
Axial T1 (**a**) and T2 (**b**) MR images of the elbow demonstrate an avulsed medial epicondyle apophysis (arrowheads) with distal distraction. There is also a low-grade partial thickness tear of the proximal UCL (solid arrows)

Stress reaction and fracture in the throwing athlete’s elbow can result from overuse and direct trauma. Oblique and transverse fractures involve the middle third of the olecranon as a result of repetitive olecranon fossa impaction from extension and valgus force, and extension force, respectively, with triceps traction during the deceleration phase of throwing [23, 25]. Medial supracondylar stress fractures have also been described and are likely secondary to a similar process of impaction and valgus load [23]. If unfused physes are present, then traction apophysitis and physeal (Salter-Harris I) fracture can occur (Fig. 11) [25]. Stress reactions manifest as localized bone marrow edema-like signal and can evolve into distinct hypointense fracture lines. As previously described, an avulsion fracture of the sublime tubercle can result from valgus force to the elbow.

Chronic osseous remodeling and osteophyte formation at sites of ligamentous traction and articular degeneration are at risk for fracture and fragmentation in the acute and chronic setting. These fractured and fragmented osteophytes, in addition to acute and chronic chondral injury, can result in intraarticular osteocartilaginous bodies, which can result in further joint damage and progressive injury. Fractured osteophytes are commonly identified in the posterior joint space in the axial and/or coronal planes. Similarly, osteocartilaginous bodies can result from displacement or fragmentation of osteochondral defects. Although somewhat dependent on size, osteophytes and osteocartilaginous bodies can be identified with radiographs and MRI. Depending on the ratio of bone to cartilage, osteocartilaginous bodies can have a variable appearance on MRI, which ranges from fatty marrow to intermediate cartilage signal. Radiographically, osteophytes are best identified on the lateral radiograph with maximal flexion.

## Conclusion

Predictable injury patterns occur in the thrower’s elbow. Pathology is typically localized to the medial aspect of the joint and can be reliably identified with imaging. Clinical history and physical examination should guide imaging evaluation. Radiographs should be the first modality of choice used to identify malalignment, fracture, arthrosis, osteochondral defects, loose bodies, joint effusion, and soft tissue calcification. Subsequent evaluation with MRI provides superior soft tissue contrast for assessment of ligaments, muscle-tendon units, and nerves. The MRI technique should be optimized to maximize spatial resolution and soft tissue contrast. The constellation of clinical, physical, and radiologic examinations should be synthesized to guide management.
